# Evidence‐based priorities of under‐served pregnant and parenting adolescents: addressing inequities through a participatory approach to contextualizing evidence syntheses

**DOI:** 10.1186/s12939-021-01458-7

**Published:** 2021-05-10

**Authors:** Anna Dion, Aime Klevor, Amy Nakajima, Neil Andersson

**Affiliations:** 1 Participatory Research at McGill (PRAM), Department of Family Medicine, McGill University, Montreal, Quebec Canada; 2 Ottawa, Canada; 3 Bruyere Continuing Care, Wabano Centre for Aboriginal Health, Consultant Gynecologist, Ottawa, Canada; 4 Centro de Investigación de Enfermedades Tropicales (CIET), Universidad Autónoma de Guerrero, Acapulco, Mexico

**Keywords:** Adolescent perinatal health, Pregnancy, Evidence synthesis, Participatory research, Stigma, Patient engagement, Patient‐oriented research

## Abstract

**Purpose:**

This study describes an interdiscursive evidence-based priority setting process with pregnant and parenting adolescents and their services providers.

**Methods:**

A mixed methods literature review identified studies reporting on perinatal outcomes and experiences of adolescents during pregnancy to 12 months post-partum published in Canada after 2000. We also calculated relative risks for common perinatal risk factors and outcomes for adolescents compared to adult populations from 2012 to 2017 based on data from a provincial database of maternal and newborn outcomes. Two trained peer researchers identified outcomes most relevant to their peers. We shared syntheses results with four service providers and 13 adolescent mothers accessing services at a community service organization, who identified and prioritized their areas of concern. We repeated the process for the identified priority issue and expanded upon it through semi-structured interviews.

**Results:**

Adolescent mothers face higher rates of poverty, abuse, anxiety and depression than do adult mothers. Adolescents prioritized the *experience of judgment* in perinatal health and social services, particularly as it contributed to them being identified as a child protection risk. Secondary priorities included loss of social support and inaccessibility of community resources. The experience of judgment in adolescent perinatal health literature was summarized around: being invisible, seen as incapable and seen as a risk. Adolescent mothers adapted these categories, emphasizing organizational and social barriers.

**Conclusions:**

Young marginalized women are disproportionately affected by inequities in perinatal outcomes, yet their perspectives are rarely centered in efforts to address these inequities. This research addresses health inequities by presenting a robust, transparent and participatory approach to priority setting as a way to better represent the perspectives of those who carry the greatest burden of health inequities in evidence syntheses. In our work, marginalized adolescent parents adapted published literature around the experience and consequences of social stigma on perinatal outcomes, shifting our understanding of root causes and possible solutions.

**Supplementary Information:**

The online version contains supplementary material available at 10.1186/s12939-021-01458-7.

## Background

Despite Canada’s investment in universal and accessible health services, people living in poorer socioeconomic conditions often have poorer outcomes than those with greater access to resources and educational opportunities [1, 2]. By international standards, Canada has a low pregnancy-related maternal mortality rate (7.4/100 000 births in 2013-14) [3]. Adolescent women in Canada have higher risk factors and poorer outcomes. Increased risks associated with adolescent pregnancies include preterm and very preterm delivery, having infants of low birth weight and/or small for gestational age, and for neonatal and infant mortality [4, 5]. Although socioeconomic and behavioural factors like smoking, alcohol and drug use, poor nutrition, and poor prenatal care are also risk factors, young maternal age remains an independent risk factor for these outcomes after adjusting for potential confounders [6]. Globally, significant resources are dedicated to reducing unplanned pregnancies in adolescence. In Canada, the age-specific birth rate among adolescents among both 15-17- and 18–19-year-olds has declined between 2009 and 2013 (from 8.2 to 5.3/100,00 live births among 15–17 year olds and from 25.8 to 18.6/100,000 live births among 18–19 year olds) [3]. Some authors suggest this is due in part to improved sexual education and increased access to contraception and abortion [4].

This paper describes our approach to contextualize available evidence in the lived experience of adolescent mothers, to identify and better to understand priority issues affecting their care as pregnant and parenting adolescents. As part of a larger initiative, this paper describes two meetings with young mothers, the first to determine the focus of the research project, and the second as an exploration of the chosen focus issue.

## Methods

### Engaging peer‐researchers

**Fig. 1:**
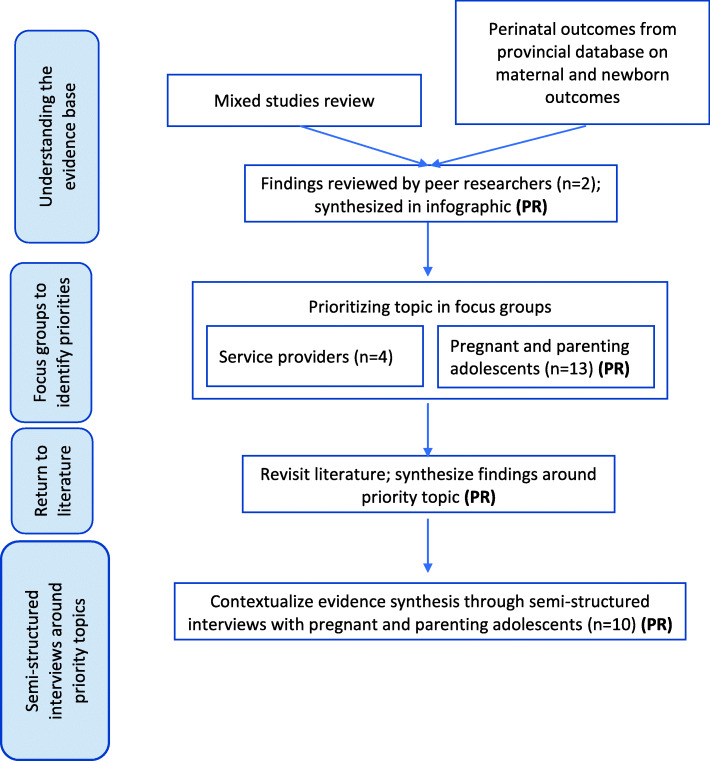
Flow chart describing the method, where PR indicates where peer-researchers were directly involved

We hired a peer researcher to work as part of this project. Two candidates were identified by staff at our partner organization, a community-based health and social service agency for young pregnant and parenting adolescents, which includes a maternity shelter for precariously housed pregnant and parenting adolescents. Both peer researchers were young mothers who accessed services at our partner organization. Both peer researchers received 10 h of peer researcher training, adapted and delivered by the lead author (AD) [7]. Peer-researchers were paid during their training and while contributing to the project. A flow chart describing the overall project methods is provided in Fig. 1 and indicates where peer researchers were actively involved.

### Mixed studies review

We searched Medline, CINAHL and Web of Science for primary research describing perinatal (pregnancy to 12 months post-partum) health outcomes and experiences of adolescent women (under 23 years of age) in Canada, including all articles published in French or English after the year 2000. We included reports of clinical outcomes and those describing the experiences of adolescents through antepartum, intrapartum and postpartum care. Where available, we also included studies describing the experience of healthcare providers caring for pregnant adolescents. We excluded clinical guidelines.

The lead author screened all abstracts, read and extracted data from all eligible articles and assessed the quality of studies using the Critical Appraisal Checklist for Qualitative Research [8]. We extracted findings using inductive thematic synthesis and descriptive statistics for quantitative data [9–11].

### Regional and provincial data on perinatal outcomes

We also analyzed data from the Better Outcomes Registry Network (BORN), a database on pregnancy, birth and childhood outcomes for the province of Ontario. We extracted data on common perinatal health indicators (such as pregnancy rates among adolescents, preterm births, access to antenatal care, labour and birth complications) as well as specific indicators commonly reported among adolescent pregnancies (substance use, sexually transmitted infections, mental health concerns, experience of abuse). We calculated relative risks for adolescents for each indicator by year and over the 5-year period for both the province and the relevant local health integration network from 2012 to 2017.

The lead author and peer-researchers reviewed statistics of outcomes and risk factors from quantitative studies along with themes, quotes and images identified from qualitative studies. We discussed which findings might be most relevant to clients accessing services at our partner agency. AD summarized selected findings in an infographic, which the peer researchers reviewed and refined.

### Participant recruitment

Wherever possible, we piloted all elements of our method with peer researchers and adopted strategies to support meaningful engagement with adolescents. Adolescent women were invited to participate in the focus groups through recruitment posters, discussion with staff of our partner organization and brief presentations by the lead author and peer researchers. We adapted consent forms to ensure that language was understandable and accessible to potential participants [12] (see Additional File A for examples). We opened each meeting with a discussion of young people’s rights when participating in research. Using a Charter of Rights for Children and Young People developed by Moore et al., we reviewed issues relating to participants’ rights to be heard, to participate in the way they prefer, to be treated well and not be hurt or discriminated against [13]. Counseling staff from our partner organization was also available if anyone needed additional support during or after participating in discussions.

 We invited women aged 16 and above to participate in our research. Many clients of our partner organization are recognized as minors withdrawn from parental control and are legally recognized as adults when engaging with service organizations. While this often adds to their vulnerability as young parents, we felt it would be inappropriate and potentially harmful to ask potential research participants under the age of 18 to seek parental consent to participate in this research project. This consideration builds on previous research on qualitative, community-based research with adolescents on their sexual health and is supported by studies suggesting that with enough time and information, adolescents over the age of 15 years have the cognitive capacity to make informed decisions [14].

 We worked with our partner organization and peer-researchers to determine appropriate honoraria for participants. We provided childcare, bus passes and snacks during each meeting to ensure that participants could engage comfortably in discussions. We also provided $30 gift cards for each 2-hour meeting in recognition of participants expertise. We distributed gift cards at the beginning of each meeting (after the consent process) so that participants did not feel obliged to stay if they were uncomfortable throughout the meeting [15]. Prior to engaging with young women as participants in this research project, the lead author (AD) was a respite volunteer for young mothers at our partner organization’s shelter for over a year before and throughout the project. Many women knew the peer researcher (AK) as a fellow client of our partner organization. This helped to build trust with participants and create familiarity with our partner organization’s activities.

### Focus groups to identify priorities

We carried out two separate focus group discussions to identify priority areas to better address pregnant and parenting adolescent needs [16]. In both focus group meetings, we presented the evidence synthesis infographic and invited participants to tour nine photos and quotes selected by peer researchers from qualitative studies. We emphasized that the findings from the literature represented how perinatal health among adolescent women was discussed in published literature and that not all aspects may resonate with their own experiences.

The first focus group was with four service providers from obstetrics, mental health, nursing and social work, all involved in providing front line services to pregnant and parenting adolescents. After reviewing the summary infographic and selected extracts from qualitative studies, they individually identified priority challenges in the perinatal health and well-being of their clients on post-it notes, and then grouped common themes between them. Each provider was given five stickers to allocate to the challenge they felt was most important to young mothers’ well-being, specifying that they could place more than one sticker per issue. Priority issues were summarized and included in the focus group with adolescents.

The second focus group was co-facilitated by the lead author (AD) and one of the peer researchers (AK). After reviewing the summary infographic and selected extracts from qualitative studies, we asked each participant to identify issues where they faced challenges or barriers throughout their pregnancy and early postpartum experience. We prompted participants to draw from the literature, priority issues identified by service providers in the first focus group, as well as their own experiences. Participants wrote their ideas on post-it notes. Where comfortable, participants presented their issue, briefly describing why it was important and placed it on the wall. As each subsequent participant shared their issue, they determined whether their issue could be grouped with one already posted on the wall or if it addressed a separate issue [17]. Any participants who did not want to present their issue themselves could hand their post-it notes to the two facilitators (AD and AK) as we circulated around the room. Before moving on to the next step, we asked participants to adjust any grouping or descriptions of their own topics if they felt their idea had changed or was miscategorised. We also included priority themes identified by service providers if they were not already mentioned for women to include in their evaluation.

Each participant received five voting stickers to identify the most important challenges faced by young mothers and clarified that they could place more than one sticker per issue. We then re-organized categories according to participants’ priorities. We gave participants an additional two stickers each, asking them to identify among those identified in the first round, their first and second priority concerns. We finalized the priority issues through group discussion, and participants were asked to write the answers to the following two questions with respect to the issue identified as the top priority: *Why is this important? What do we still need to know about this?* [7]

### Return to literature

 We reviewed the studies included in our mixed review to re-assess how these studies explored the issue identified during the focus group. The lead author (AD) identified primary and second-order themes related to the priority issue. Second-order themes are grounded in evidence from the original studies but are the result of identifying patterns or central ideas across the collection of studies [18]. These themes were further refined by the peer researcher (AK).

### Semi‐structured interviews

We used the themes to guide individual or small group discussions with 10 young mothers, where they generated their own ideas and then arranged them around the second-order themes identified in the literature, creating new categories when needed. They subsequently incorporated primary themes from the literature that they felt were relevant to their own experience. The lead author and peer researcher independently reviewed the concept maps and notes from each of the interviews and developed a list of common themes. They compared themes and refined them to reach a final set of themes grounded in the experience of participating women.

 Ethics approval was received from the McGill Faculty of Medicine Ethics Review Board (A09-B51-17 A). An Advisory Board made of senior staff of our partner organization also refined and approved this research.

## Results

### Mixed studies review

**Fig. 2:**
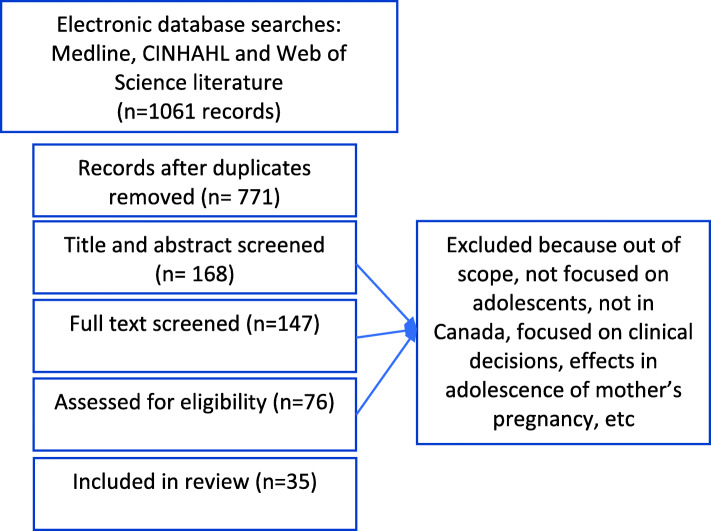
Flow chart describing the screening of articles in mixed studies review of adolescent perinatal outcomes in Canada

Our search identified 771 publications. The lead author (AD) assessed all abstracts to determine eligibility and extracted data from 35 relevant articles (24 quantitative, 11 qualitative). A flow chart of our review process is shown in Fig. 2.

Adolescents with major mental illness have a higher age-specific fertility rates than adults with major mental illness. [19]. Pregnant and/or parenting adolescents were two to four times more likely ever to have experienced physical abuse [20], were more likely to be single and were four times more likely to have a low income (less than $40 000 a year) [21]. Roughly 60 % of young mothers accessing care at a youth pregnancy outreach clinic either completed or were in the process of completing high school [22]. Pregnant adolescents were 10–47 % less likely to attend prenatal care in the first trimester, often citing financial barriers, long waiting times, lack of privacy, fear of judgment and not wanting to miss school [4].

Themes identified in qualitative studies reflect the complexity of emotions surrounding pregnancy and motherhood. Themes and illustrative quotes are shown in Table 1. They include gratitude for their experience of pregnancy and their children, while also experiencing pervasive social stigma navigating education, employment and social services for themselves and their children [23–26].

**Table 1:** Themes Identified in Qualitative Literature

Theme	Illustrative Quote
**Motherhood as Transformative**	*“I was pregnant, and realized the path that I was going to bring another human being into. This was my choice – so there were two paths for me to go on. To continue going on this one and bring a child in that, not being in control of my life. And I knew that I couldn’t do that to a child. So, making the choice to go the straight path and know what is coming.”* [25]
**Judgment**	*”My social worker questioned my ability to mother properly.”* [26]
	*”It bothers me what other people think. I am trying really hard not to think about what other people think about my mothering. Do you know what I mean? … Because everyone is telling us that we can’t*.*”* [25]
**Control**	*“And actually I had a friend that took something for the birth and it showed [in a subsequent drug test] that she did drugs, when she’s not that kind of person. And, they instantly took that baby … So, I didn’t take anything [during labour]. It was pretty crazy. I was scared.”* [26]
**Need for Comprehensive Support**	“*I am not ashamed of being a teen mother. However I do feel that if someone had guided me when I was going through my eating disorder, addictions, and insecurities that my life could have been different.”* [23]
**Poverty and Meeting Basic Needs**	*“Umm, just healthy foods. I find that they’re really hard to access. That ties in really huge with women’s health right?”* [23]
	*“if you don’t have a safe place to call home, then you’re not going to be able to get any other supports for yourself in place including anything for your sexual health*.*”* [23]

### Data from provincial database

Ontario-specific findings were similar to those reported in the literature. Adolescent mothers in Ontario were more likely than adult mothers to have a mental health diagnosis of anxiety (relative risk (RR) = 1.77, 95 % CI 1.72–1.81), depression (RR = 2.16, 95 % CI 2.11–2.22) as well as more severe mental health disorders (RR = 2.88, 95 % CI 2.75-3.00). Pregnant and/or parenting adolescents were more likely to have used illegal substances (RR = 5.63, 95 % CI 5.41–5.84) or alcohol (RR = 2.33, 95 % CI 2.22–2.44) during pregnancy and they were more likely to be diagnosed with a sexually transmitted infection during pregnancy (RR = 2.77, 95 % CI 2.62–2.93).

 Evidence tables from our review as well as risks and outcomes among adolescent (under 23 years) and adult pregnancies in the Champlain Local Health Integration Network and across Ontario extracted from the BORN database are in Additional File B.

**Fig. 3:**
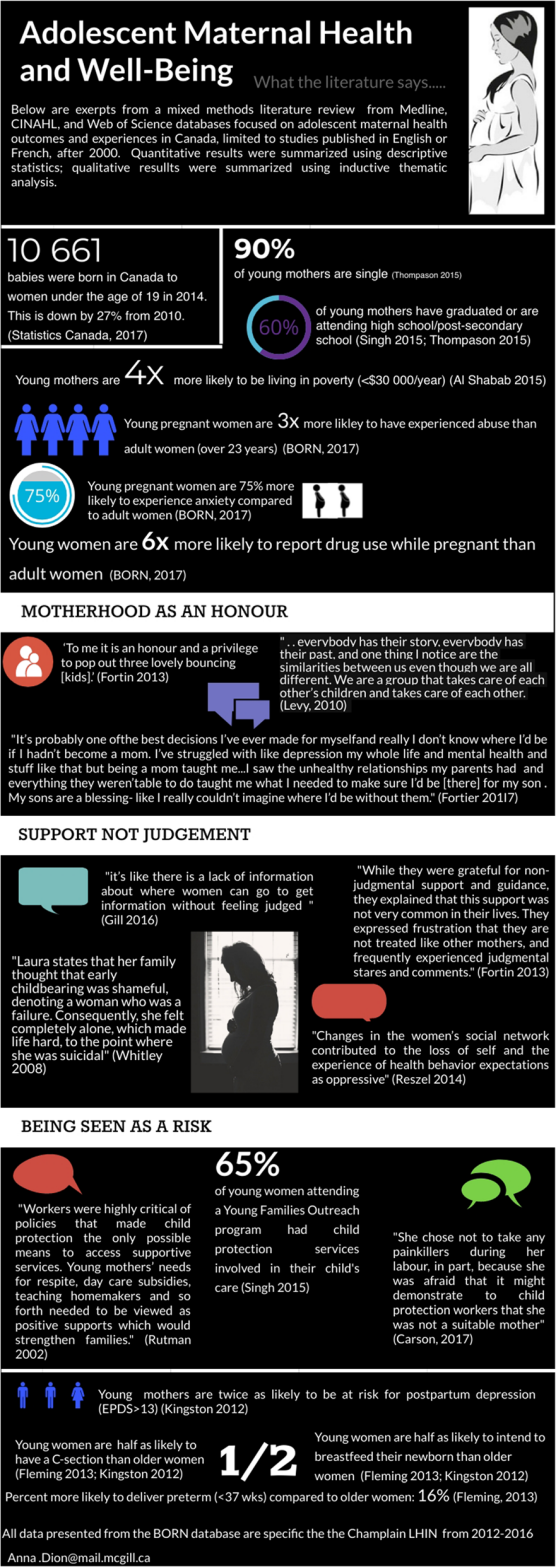
Evidence Synthesis Infographic describing findings from a mixed methods literature review of perinatal outcomes and experiences of adolescents in Canada from 2000-2019. Infographic was co-developed with peer researchers involved in this study

We finalized the evidence infographic (Fig. 3) and peer researchers identified nine quotes and photographs from four qualitative studies that used PhotoVoice methodologies identified through our review to include in priority setting focus groups with service providers and young women [23, 25, 27, 28].

.

### Focus group to identify priorities

Four service providers from the fields of obstetrics, mental health, nursing and social work, all involved in providing front line services to pregnant and parenting young people (under the age of 25) in Ottawa, contributed to identification of service priorities. They pointed to the need for access to mental health services and to the influence of stigma and fear of the consequences of being vulnerable as critical factors influencing perinatal well-being, as outlined in Table 2. They saw young women’s on-going precarity due to poverty, housing instability and the need for independent living skills as factors contributing poorer perinatal outcomes, highlighting the need for trauma-informed and culturally informed programming.

**Table 2:** Priority Areas to Support the Well-being of Pregnant and Parenting Youth, Identified by Service Providers and Young Women

Priority Areas Identified by Service Providers	Priority Areas Identified by Young Women
**Mental Health**	**Judgment**
• Access to mental health services	• In being identified as a child protection risk
• Anxiety and Depression	• Accessing housing
• Untreated or undiagnosed mental health needs	• Breastfeeding
**Judgment**	• Accessing health services
• Fear of being vulnerable; fear of being flagged as a risk to Child Protection	**Lack of connection with supports**
**Past Traumatic Experiences**	• Loss of social support network/Isolation
• Impact of childhood trauma on parenting	• Not knowing options and resources
• Impact of domestic violence; unstable relationship with child’s father (or mother)	**Navigating Care/Institutional Barriers**
**Safe and Supported Living**	• Access to mental health services
• Unstable inadequate unaffordable housing	• Expectations
• Access cultural perspectives towards parenting and perinatal care	• Permanently labeled
• Lack of life skills to support independent living	**Influence of childhood trauma on parenting**
• Poverty’s impact on accessing care	**Intimate Partner Violence**
**Lack of coordinated services for youth**	**Poverty**

Thirteen women aged 17–25 years participated in the second focus group. Participants’ children ranged in age from one month to 4 years old, with between 1 and 4 children per woman, and women had varying levels of custody of their children. As outlined in Table 3, women overwhelmingly identified the experience of being judged or misunderstood as their most important challenge throughout their maternity and early motherhood experiences. Women experienced judgment in everyday experiences, such as on public transportation or when grocery shopping, which eroded their sense of confidence. Many women reported receiving negative comments, including being asked to leave public spaces while breastfeeding their infants in public.

Young moms and moms in general are still constantly being shamed and ridiculed for breastfeeding in public…it makes me mad when I see women being shamed for it. If you don’t like to see it, look the other way.

After discussing women’s experiences of judgment across multiple areas of their lives, women identified being identified as a child protection risk as their most important concern. Several women stated that their interactions with child protection workers themselves had been generally positive, but that initial reports to the Children’s Aid Society’s were uninformed or made without adequate investigation or contextual understanding. Women sought to be seen for their strengths as well as challenges, despite and not because of their age, and emphasized early and non-judgmental support to prevent the need for child protection involvement. This priority area was further explored with participants in subsequent meetings and described in a sister publication.

*Return to the literature*: Upon reviewing the studies identified in our literature review, we identified three second-order themes describing how judgment is experienced by pregnant and parenting adolescents: *being seen as a risk, being seen as incapable* or not being seen at all (*being invisible).* First-order themes that contributed to each of these themes are shown in the left-hand column of Table 3.

### Semi‐structured interviews

 Ten women participated in follow-up interviews where all but three had participated in the focus group meeting described above. We held these interviews individually or in groups of two depending on participant preference. After describing their own experiences of judgment during pregnancy and postpartum period, all participants confirmed the relevance of the second-order themes. Participants organized themes describing their own experiences and selected first-order themes from the literature under each of the second-order theme, and described the consequences or effects of each type of judgment. Some participants described experiences or events but were uncomfortable with formally documenting them. We supported the distilling of ideas, but participants decided how their experiences were described, represented or even included, emphasizing that participants also had the right to not share their stories [29]. We describe how participants adapted and contributed to each of the second-order themes below, summarized in Table 3.

**Table 3:** First and second-order themes identified in the literature, with additional themes added by participants around the experience of judgment and its consequences for perinatal health

**Experiences of Judgment**		
**Second-order theme: Seen as a Risk** *First order themes identified in literature* • Blamed for things outside of control [30–33] • Pressure to show “good motherhood” [24, 26, 31–33] • Asking for help leads to blame [24, 30, 34] • Always under microscope [25]		*Themes added by women* • Using my past against me • Seen as dangerous if I stand up for myself
**Second-order theme: Seen as Incapable** *First-order themes identified in literature* • Assumed incompetence [25, 32] • Consent not seen as necessary [30, 32]		*Concepts added by women* • Loss of confidence of others; affects belief in oneself
**Second-order theme: Invisible** *Primary themes identified in literature* • Own needs not recognized [23, 34]		
** Consequences of Judgment**		
*First-order themes identified in literature* • Internalized blame [30, 35] • Self-doubt [30, 31] • Unmet needs [23, 30, 33, 34] • No voice in decision-making [25, 33]		*Concepts added by women* • Anger, Frustration • Contributes to anxiety and depression • Must fight to be heard • Makes me want to give up

#### Seen as a risk

Women described feeling discounted or quickly judged by service providers who did not understand the broader context of women’s experiences. Women were frustrated by experiences of surveillance and judgment rather than support when they disclosed needs around housing, low income, or other health and social concerns.

They judge you for something you didn’t even know you are doing. They make you feel like you are not capable of being a good mom….instead they should try to help you become the best mom you can be.

Women felt they did not have enough information about programs and services available to them, particularly those focused on the needs of pregnant and/or parenting adolescents. Several women reported avoiding services or not fully disclosing needs for fear of judgment. This left them with unmet needs, particularly around mental health.

I also had to balance not saying too much. I couldn’t say what I really feel for fear that it would be seen as a risk to my child, and would be reason to justify her removal or the removal of my rights.

Women reported feeling let down or judged by those they thought could support them; in many cases, losing social support from friends, partners and sometimes family, upon learning they were pregnant. Women often spoke about isolation and harassment from friends at school or work, unsupportive teachers and/or family members who may disapprove of their pregnancy and/or their partners.

It is judgment from the people that have meant something to me that hurts the most

Women also described feeling permanently labeled based on their needs or events in their lives, over which they had little control. Examples included parenting with a disability, having had child protection involvement in their own childhood or by what were seen as choices to remain in abusive or violent relationships.

I have a mild form of autism, so that brought additional judgment. They were telling me that I shouldn’t be having kids because … neither of us will know what to do with a child.

We don’t need to be hovered over -- we aren’t terrible or scary people, or that we have no idea what we are doing. Some of us are in bad situations or made some poor choices along the way, but it doesn’t mean we don’t know anything.

Women described having to counter ideas around parenting norms particularly related to the absence of partners or extended family in their lives. For them, isolation from these relationships was difficult but often necessary.

Then she [my child’s doctor] began to be really opinionated about me needing to get back together with my son’s dad. Eventually, I told her that I didn’t want to go back to being beaten every day and she backed off… Isolation is seen as a bad thing, but when your support network isn’t good for you, sometimes it is the best thing.

#### Invisible

Several women discussed the paradox of navigating health and social service systems as a young mother, where they were expected to manage the responsibilities of new motherhood as an adult, while still being a minor, from both a legal and societal perspectives. In the words of one participant *“I am old enough to have a baby….but not old enough to give consent [for my own medical needs]?!?”*,

#### Seen as incapable

Women linked experiences of social stigma with higher levels of stress, frustration and in some cases anger. They reported feeling as though they were made to feel incompetent before being given a chance.

When I had my first child in the hospital, people just took over, with the assumption that I wasn’t going to do it myself.

I sneeze and it is judged; I go to the bathroom and I wonder if it is okay…..you cannot function as a human if you are always in doubt

These experiences contributed to women feeling as if they had no voice in or control over their care, undermining their position as primary caregivers. Some women reported feeling that their consent in the care and handling of their child was not respected, with one woman limiting her sleep in the hospital both ante and post-partum for fear that decisions about her newborn’s care would be made without her.

#### Refusing judgment

Women refuted judgment and its consequences by affirming their identities as mothers. They invested considerable mental and emotional energy in controlling the narrative in how they were perceived, learning to advocate for themselves and their children early on.

You can either deal with judgment and live your life, or hide away. I hid for a while but then I decided I just didn’t care what other people think, but I had to grow up fast to get there.

Women also reported refusing support that did not meet their needs, which they felt resulted in being labelled as non-compliant.

I wasn’t interested in participating in an arts and crafts program but had to, as well as other programming that I didn’t find helpful. I pushed back against the rules because I didn’t feel they were helpful or what I needed.

“I am generally uncomfortable with male authority figures and didn’t want a man examining my baby, so when the male resident came to examine my daughter, I refused the exam.”

Women also emphasized the importance of supportive relationships in preventing difficult circumstances from evolving into more serious risks for themselves and their children. This often included family members and close friends, as well as health and social service providers, particularly those working within adolescent-specific services.

For me it was my mum and grandmother - telling me that I am a good person, that I can do this. They made me believe in myself.

There were some good people at the hospital- they showed that they had faith in me and took the time to spend some time with me. One was a lactation consultant, who stood up for me within the hospital and with other professionals.

## Discussion

This research summarizes available published evidence around adolescent pregnancy in Canada and describes how this evidence is prioritized and understood by adolescent mothers themselves. Adolescent mothers face higher rates of poverty, abuse, anxiety and depression than do adult mothers. Adolescents prioritized the *experience of judgment* in perinatal health and social services, particularly as it contributed to them being identified as a child protection risk. Women’s experiences of judgment around pregnancy and parenthood had important implications for mental health, their identity as mothers, and access to services. They emphasized the importance of supportive relationships and their role as advocates to counter the consequences of judgment.

Social norms around motherhood play a large role in the experience of adolescent motherhood [36–38]. Women described barriers specific to young parents that are built into the structure and organization of institutions. These barriers affected women’s access to health, opportunities for education, financial support, housing and in navigating child protection issues, exacerbating existing vulnerabilities, even in the absence of individual prejudice or discrimination on the part of care providers [39].

Experiencing repeated signals of inadequacy often leads to internalizing of negative stereotypes, and can influence people’s willingness to seek care, as well as how care is acted upon and what is refused [29, 40]. Fearing rejection, women guarded against or avoided potentially threatening interactions altogether. Women in our study mentioned the mental health consequences of repeatedly feeling judged, having their identity as mothers undermined or questioned, and the invisible emotional work to manage how service providers perceived them.

Women found themselves labeled as ‘non-compliant’ when they did not access the care system that does not adequately consider their needs. When policies and resource allocation does not align with community needs, however, providers might also lack the support of strong inter-professional collaborations to provide integrated and community-based perinatal care [41–43]. In a matched cohort study, Fleming et al. found adolescents receiving specialized multidisciplinary community-based perinatal care had significantly lower risks of low birth weight and preterm delivery, and higher rates of prenatal visits, prenatal class attendance and group B streptococcus screening compared with adolescents across Ontario, despite higher levels of tobacco, alcohol and other substance use than the control group [44].

These findings have several implications for research and for the provision of perinatal care for young people. Researchers, clinicians and care providers can lessen the extent to which judgement shapes maternity and early parenthood experiences, especially among those who may face high levels of stigma. We need interventions to shift the deeply held attitudes and beliefs that lead to labelling, devaluing and discriminating and the processes that maintain these perceptions as dominant ones [40, 45]. Recognizing the value of lived experience in informing service delivery can strengthen our understanding of the influence of social and organizational contexts in health interventions [46]. This is particularly important in stigmatised communities, where incorrect assumptions or representations may reinforce negative stereotypes [47].

The interdiscursive approach we describe in this article is a systematic yet simple approach to grounding evidence in local contexts with a population that has had limited opportunities to become familiar with evidence synthesis approaches. We carried out this evidence-based priority setting exercise to determine priority areas for a subsequent stage of research, and therefore we considered the perspectives of local stakeholders as contributing contextual knowledge that was important in identifying priority topics. This does not diminish the important contributions of biomedical and other forms of research or suggest that one type of knowledge has a hierarchy over others. Rather we suggest that people make better decisions when they benefit from both evidence-based perspectives, meaning those transferred through theoretical or statistical inferences, often developed through primary studies or syntheses, as well as context-specific understanding, based on local settings, experience and tacit understanding of practice and organizational ‘know how” [48]. By valuing the voices of pregnant and parenting young people in determining the focus of our research, we were able to focus discussions on what mattered most to participants. This probably also increased engagement and participation in subsequent stages of our research. Inviting stakeholders to engage with the full scope of evidence often available to other decision-makers and subsequently identify priorities is an important mechanism to prevent the dismissal of community or informal knowledge on the grounds of not having full understanding of an issue. Our findings are particularly relevant for local service improvements and point to additional areas to consider for future evidence syntheses.

## Conclusions

Centering knowledge synthesis on adolescent women’s voices changed the focus of our research. An iterative process grounded conventional evidence in young women’s lived experience, deepening our understanding of the role of judgment in shaping perinatal care. Women’s experiences of judgment around pregnancy and parenthood had important implications for mental health, their identity as mothers, and access to services. They emphasized the importance of supportive relationships and their role as advocates to counter the consequences of judgment.

 Adolescent parents are disproportionately affected by inequities in perinatal outcomes, yet their perspectives are rarely heard in efforts to address these inequities. We used transparent and participatory methods to strengthen the voice of marginalized adolescent parents in identifying clinical and research priorities that address their needs. Without meaningfully involving those most affected by an issue, we risk leaving already marginalized groups underserved and further excluded from the benefits of care and quality improvement initiatives.

## Supplementary Information

**Additional file 1.**

**Additional file 2.**

## Data Availability

All data generated or analysed during this study are included in this published article and its supplementary information files.
