# Supplementary material for: Evidence‐based priorities of under‐served pregnant and parenting adolescents: addressing inequities through a participatory approach to contextualizing evidence syntheses
Source: Int J Equity Health. 2021 May 10;20:118. doi: 10.1186/s12939-021-01458-7 (PMC8111962; doi:10.1186/s12939-021-01458-7)
Supplement: Supplementary file 1 — Additional file 1. [file 12939_2021_1458_MOESM1_ESM.pdf]

Mapping Our Understanding of Maternal Health

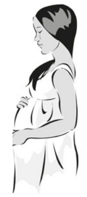

What is this project about?
It is about better understanding the reasons for challenges faced by young pregnant and/or parenting people to improve health and social services before and after pregnancy.

Why am I invited?
We are inviting you to participate in this project because we want to hear what is important to you as a young parent. We also want to ask what your ideas are for solutions to common issues faced by young pregnant and parenting people.

Who is running this project?
This project is run by Anna Dion, a PhD student at McGill University, and has been developed under the supervision of Dr. Neil Andersson, and in partnership with staff of XXXX. This research is supported by a scholarship from the Pierre Elliott Trudeau Foundation.

What do I have to do?
We are asking you to participate in 4 meetings. Each meeting will last about 2 hours and will take place between May and November 2018.

At the first meeting (on Wednesday May 30th from 1:00-3:00pm)
We will share some information about health issues that are common in people who are young and pregnant and/or parenting. In a group with other young women, we will ask you to tell us which ones are most important to you and why.

At the second meeting (in June and July):
In a meeting with the researcher (Anna Dion), we will ask you to tell us the reasons why you think a specific health problem is common in people who are young and pregnant. We will ask you a series of questions about your experience as a person who is young, pregnant and/or parenting and ask you to make a map of your ideas with magnets, markers and a magnetic white board (which we will give to you). We will also ask what reasons you think are most important. You will be able to choose whether you participate in this meeting with others or by yourself.

At the third and fourth meetings (in September and October):
In a group with 6-8 other women, we will share some possible reasons to explain why a specific health issue might be common in people who are young and pregnant or parenting. We will ask what you think about these explanations and how they relate to your own experience. We will also ask for your ideas about how to improve services before and after pregnancy for young people. We will also share some suggestions from others groups of people and ask what you think of these suggestions.

What will happen to the information?
This information will be shared with organizations involved in supporting young pregnant and parenting people in XXXX and across Canada. Some of the information will be written in reports,

articles and presented at conferences. None of your personal or identifying information will be shared through any of these.

Will it be private?
Yes. You do not need to give your name or you can make one up to use while you are participating in this project. Once the project is finished, there will be no way to connect you with the information you share through this project.

While we ask that everyone participating in the group discussions respects the privacy of others, it is possible that they may not.

Do I have to participate?
No. You can choose not to participate. No one will be mad or upset with you if you don't. You will still be able to come to all of the same programs at XXXXX. You can also decide not to participate at any point in this project. If you decide to stop participating, you can also ask that your map of ideas not be included in this study.

Will I get anything if I participate?
Yes. You will receive a gift card for Walmart for $25.00 after each of the 2-hour meetings, for a total of 4 gift cards over the course of the study. If you decide not to participate in all of the meetings, you will receive gift cards for the meetings you attend.

Childcare will also be provided by the regular childcare staff at XXXXX during all meetings. We will also give a Presto Card with pre-paid trips on it to cover your costs of getting to and from the meeting by bus. There will also be light snacks and drinks available during all meetings.

Are there any risks to participating?
It is possible that you may feel uncomfortable discussing during some of the meetings if it causes you to remember unpleasant or painful experiences. You are free to not participate in any discussion. You are also free to leave at any time. We will have a place in our meeting rooms for you to have some personal space and XXXXX staff will be available during all of our activities if you would like to speak with anyone about your feelings.

Who can I ask if I have questions about the Mapping Our Understanding project?
You are welcome to ask questions before, during, or after your participation in this research. Please contact:

Mapping Our Understanding of Maternal Health

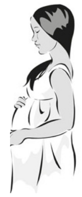

Meeting 1: Identifying Focus Areas: May 30th 2018
1:00-3:30pm

I understand that:	Please check:
A researcher, Anna Dion, with up to two peer facilitators, will come to XXX and run a group discussion with me and other young people. We will spend roughly 2.5 hours together.	
They will share some information about health issues that are common in people who are young and pregnant and/or parenting and we will talk about what issues are most important to us.	
I don't have to answer questions that I don't like or don't want to answer. I also don't have to participate in group discussions if I don't want to.	
I can choose to stop participating in the meeting or the whole project at any time without giving any reasons.	
If anything we talk about makes me feel upset, I can take a break from the discussion or leave the meeting. I will be given the names of people who I can talk to about what is making me upset.	
What I say during project meetings and interviews is special and belongs to me. The researcher or peer facilitators won't tell anyone else that I participated in this project. They will ask everyone in the group to agree not to talk about what is said during project meetings unless all of us say that it is okay.	
What I say during this meeting might be used in a report or presentation, but the researcher will make sure that nobody will be able to tell who I am or what I said.	
The only time the researcher would have to tell someone about anything I said is if they were worried:<br>• that I or my child(ren) might be badly hurt by someone<br>• that I might hurt myself<br>• that I might hurt someone else.<br>The researcher will talk to me about this and I will have a say in deciding what happens next.	
I will be given a copy of this form to take home with me.	
I have been able to ask questions about this project and meeting. I am satisfied with the answers to my questions. I understand that it is okay for me to ask questions at any time if I don't understand anything.	

Participant Signature ____________________ Date ____________________

Researcher's Signature:

I have explained this study to the best of my ability. I invited questions and gave answers. I believe that the participant fully understands what is involved in being in the study, any potential risks of the study and that he or she has freely chosen to be in the study.

Researcher Signature ____________________ Date ____________________

Meeting 2 Consent v1 April 2018

Mapping Our Understanding of Maternal Health

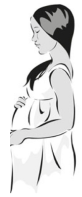

Meeting 2: Mapping Evidence: Date July 25, 2018
1:00-3:00
Program Room C

I understand that:	Please check:
A researcher, Anna Dion, will come to XXX and interview me about topic my experience of feeling judged throughout maternity care	
They will ask me why this topic is important to people who are young and pregnant or parenting. They will ask me a series of questions about my experience and will ask me to make a map of my ideas with magnets, markers and a magnetic white board. They will also ask me to identify the reasons I think are most important. I will be able to choose whether you participate in this meeting with others or by myself.	
I don't have to answer questions that I don't like or don't want to answer. I also don't have to participate in the interview if I don't want to.	
I can choose to stop participating in the meeting or the whole project at any time without giving any reasons.	
If anything we talk about makes me feel upset, I can take a break from the discussion or leave the interview. I will be given the names of people who I can talk to about what is making me upset.	
What I say during project meetings and interviews is special and belongs to me. The researcher or peer facilitators won't tell anyone else that I participated in this project.	
What I say during this interview, and the map that I create, might be used in a report or presentation, but the researcher will make sure that nobody will be able to tell who I am or what I said.	
The only time the researcher would have to tell someone about anything I said is if they were worried:<br>• that I or my child(ren) might be hurt by someone<br>• that I might hurt myself<br>• that I might hurt someone else.<br>The researcher will talk to me about this and I will have a say in deciding what happens next.	
I will be given a copy of this form to take home with me.	
I have been able to ask questions about this project and interview. I am satisfied with the answers to my questions. I understand that it is okay for me to ask questions at any time if I don't understand anything.	

____________________ ____________________

Participant Signature Date

Researcher's Signature:

I have explained this study and answered questions to the best of my ability. I believe that the participant fully understands what is involved in being in the study, any potential risks of the study and that he or she has freely chosen to be in the study.

____________________ ____________________

Researcher Signature Date
