# Supplementary material for: Evidence‐based priorities of under‐served pregnant and parenting adolescents: addressing inequities through a participatory approach to contextualizing evidence syntheses
Source: Int J Equity Health. 2021 May 10;20:118. doi: 10.1186/s12939-021-01458-7 (PMC8111962; doi:10.1186/s12939-021-01458-7)
Supplement: Supplementary file 2 — Additional file 2. [file 12939_2021_1458_MOESM2_ESM.pdf]

Evidence-based priorities of under-served pregnant and parenting adolescents: Addressing inequities through a participatory approach to contextualizing evidence syntheses
ADDITIONAL FILE B: Evidence tables from literature review (qualitative and quantitative), and analysis from provincial maternal and newborn data registry

ADDITIONAL FILE B.1 Data extraction tables from literature review on experiences and outcomes of adolescent pregnancies in Canada (1980-2018)
Adolescent Outcomes

**Author**	**Study Type**	**Location**	**Year**	**Main Objectives**	**Inclusion Criteria**	**Comparison**	**N**	Pre-eclampsia	**Gestational Diabetes**	**Preterm Delivery**	**Extreme Preterm Delivery**	**SGA/IUGR**	**LBW**	**LGA**	**Have vaginal delivery**	**Induction of Labour**	**C/S**	**Instrumental Delivery**	**Epidural**	**Rate of Prenatal Attendance**	**Prenatal Visits in First Trimester**	**Intention to Breastfeed**	**Did not initiative any b/f**	**Rate of Exclusive b/f @ discharge**	**Maternal hospitalization on poatpartum**	Rate of Anomalies	EPDS above 13	Admitted to NICU	**Fetal Death**	**Child Protection Involvement with the child**
Fleming, 2013	Cohort	Ontario	Jan 2006-Dec31 2010	Characterize the association between adolescent pregnancy and adverse perinatal, obstetric and neonatal outcomes in comparison to adult women	Singleton birth (~24000 records)	Adult deliveries	~24000	aRR0.91 (0.83-1.00)	aRR=0.34 (0.3-0.39)	aRR1.16 (1.02-1.31		aRR=1.00, (0.96-1.04	aRR=1.05 (1.00-1.11		aRR=1.76 (1.7-1.82)	aRR=1.05 (1.02-1.08)	aRR=0.57 (0.55-0.59)	aRR=0.76 (0.73-0.79)	aRR=0.93 (0.91-0.96)	aRR=0.87 (0.85-0.91)	aRR=0.53 (0.51-0.55)	aRR=0.57 (0.54-0.59)		aRR=0.73 (0.71-0.76))				aRR=1.08 (1.02-1.14)	aRR=1.02 (0.88-1.18)	
Kingston, 2012	Cross-sectional study	Canada	2006	Compare the maternity experiences, knowledge and behaviours of Canadian adolescent (15-19), young adult(20-24) and adult women (>25yrs)	Mothers who were at least 15 years of age, who gave birth in Canada to singleton infantsand who were living with their infants at the time of the interview (6421 women in total; 167 adolescents) (from the MES)	young adult(20-24) and adult women (>25yrs) Results reported for comparison to adults (+25y)	73797										aOR=0.64 (0.46-0.9)					aOR=1.63 (1.2-2.20)	aOR=2.55 (1.76-3.71)				aOR=2.29 (1.48-3.54)			
Shrim, 2011	Cross-sectional study	Montreal	2001-2007	To determine if and what type of obstetrical outcomes are more prevalent among adolescents compared to adult women at a Canadian tertiary level hospital	Singleton pregnancies to nulliparous women; divided by age (<20 and 20-39yrs)	Women who delivered in MUHC hospital who were 20-39yrs	9744			OR=2.3 (1.6-3.2)	OR=4.5 (2.5-8.1)	OR=2.4 (1.6-3.5)	OR=3.7 (2.2-6.1)				OR=0.3 (0.2-0.5))	OR=0.1 (0.05-0.4)								OR=1.8 (1.2-2.6)		OR=2.1 (1.5-3)	OR= 3.8 (1.9-7.5)	
Kim, 2015	Cross-sectional study (MES study)	Canada		To study the association of social support received during pregnanct and after childbirth on PPD; identfiy if this relationship is different for teen mothers (15-19 years) than to adult mothers (20+ years)	Mothers who were at least 15 years of age, who gave birth in Canada to singleton infantsand who were living with their infants at the time of the interview (6421 women in total; 167 adolescents)	Adult deliveries	6421 (of which 288 were teenage mums)																		teen mum =8% (n=23); adult mums: 3.1% (n=188)		14% of teen mums (n=288) vs 7.2% of adult mums			
Leclair, 2014	Retrospective cohort study	Ontario	2006-2012	to determine the association between multiple high-risk characteristics of pregnant adolescents with intention and initiation of breastfeeding	adolescent women (younger than 20 years) with a singleton live-born infant at term gestation (37 weeks or greater) from the BORN registry		22023								72%		16.40%	11.60%						48%						
**Adolescents Outrech Clinic/At-Risk Adolescents**																														
Singh, 2015	Cohort	Toronto	2011-2014	Establish a dynamic patient database allowing demographic data and outcome measures to be evaluated	High-risk ado mums and infants receiving care through Young Families Program throug Sick Kids	N/A	90																30%							65%
Fleming, 2012	Matched cohort study	Ontario	April 2004-March 2010	Determine if adolescent women followed in community-based adolescent outreach program had improved perinatal outcomes compared with matched provincial controls	Women <20yrs who received care through outreach program, matched by maternal age, year of delivery and birth volume of hospital in provincial database (N=204 cases, 831 controls)	Adolescents across province	looks at at-risk youth, but no outcomes reported	i: 2.9% i:4%	**I:0.6% c: 1%**	i: 7.3% c: 11.1%; aRR=0.47 (0.22-1.00)	i: 0.5% c: 2.5 (before 32 weeks)	i: 11.2%; c: 13%	less than 2500 g i: 5.9% c: 9,2%; less than 1500 g: i:0.5% c: 2.3%; aRR=0.41(0.18-0.95)	i: 7.8%c: 5.2	i: 90.8% c: 86.3%	i: 21.8%; c: 24.9%	i:9.2% c: 13.7%		i:80.3% c: 73.5%	attended 1st trim visit: i: 76.7%; c: 64%	aRR=1.25 (1.13-1.39) (controlling for age, smok, drugor alc use, hypertension)	int: 82.8% c: 83.1%		i: 40.7%; c: 45.6%		i: 3.4% c:4.1%				
Thompson, 2015	Retrospective cohort study	Toronto	2005-2009	to describe a population of adolescent parents and their children assessed in a Canadian urban hospital-based clinic to better delineate the clinical needs of this high-risk patient population, with a goal to optimize short- and long-term outcomes	Research subjects were recruited from The Young Families Program, an outpatient interprofessional clinical program within the Division of Adolescent Medicine in an urban, tertiary care academic children's hospital in Toronto, Ontario. The Young Families Program serves youth 12 to 18 years of age, and their children from birth to two years of age.	none	N=116(but varies by question category)											20% (n=65)							19% (n=42)					

ADDITIONAL FILE B.2 Pregnancy risks and outcomes in adolescent and adult pregnancies in the Champlain Local Health Integration Network and across Ontario, 2012-2017.

	2012-2017			
	Champlain LHIN		Ontario	
	RR	95% CI	RR	95% CI
Self-report drug and substance use in pregnancy	6.34	5.63-7.15	5.63	5.41-5.84
Self-report alcohol exposure in pregnancy	1.38	1.18-1.59	2.33	2.22-2.44
Reporting mental health anxiety*	1.76	1.62-1.9	1.77	1.72-1.81
Reporting mental health depression*	2.05	1.88-2.24	2.16	2.11-2.22
Reporting other mental health conditions* (bipolar disorder, schizophrenia, and others)	3.05	2.65-3.52	2.88	2.75-3.00
Disclose abuse during pregnancy	3.18	2.75-3.68	2.76	2.64-2.90
Did not attend prenatal care in 1st trimester**	1.11	1.10-1.12	1.10	1.094-1.098
Sexually transmitted infection during pregnancy*** (chlamydia, gonorrhea, syphilis, HSV, HIV, HPV)	1.98	1.59-2.46	2.77	2.62-2.92
Preterm Delivery (<37wks)	1.11	0.99-1.24	1.07	1.03-1.10
Labour and birth complications (Atypical or abnormal fetal surveillance)	0.99	0.93-1.06	1.00	0.982-1.024
Obstetrical Complications (IUGR/SGA, preterm delivery, UTI)	1.51	1.35-1.68	1.44	1.39-1.49

*Concerns during this pregnancy including those pre-existing, diagnosed during pregnancy or active during pregnancy. Both diagnosed or self reported;

**Before 13 weeks gestation

***Infection identified during pregnancy
